# Validation of a short turnaround time automated method for the 24/7 determination of plasma d-lactate on Roche Cobas c502

**DOI:** 10.1016/j.plabm.2023.e00317

**Published:** 2023-06-19

**Authors:** Adrien Turban, Sophie Gaubert, David Luque-Paz, Céline René, Nicolas Collet, Maxime Pawlowski, Claude Bendavid, Charles R. Lefèvre

**Affiliations:** Laboratoire de Bactériologie, Centre Hospitalier Universitaire de Rennes, F-35033, Rennes, France; Laboratoire de Biochimie-Toxicologie, Centre Hospitalier Universitaire de Rennes, F-35033, Rennes, France; Service des Maladies Infectieuses et Réanimation Médicale, Centre Hospitalier Universitaire de Rennes, F-35033, Rennes, France; Laboratoire de Biochimie-Toxicologie, Centre Hospitalier Universitaire de Rennes, F-35033, Rennes, France

Dear editor:

d-lactate is the dextrogyre enantiomer of 2-hydroxypropanoic acid and a highly specific metabolite of bacterial anaerobic glycolysis. It is also marginally produced (reference range: 0–10 µmol.L^−1^ in plasma [1]) in human metabolism by the mitochondrial d-lactate dehydrogenase (D-LDH; enzyme commission number: EC 1.1.1.28) [2,3] and detoxified in the methylglyoxal pathway [4]. It was first shown that this biomarker was elevated in short bowel syndrome [5], but it has recently been highlighted too in broad gastrointestinal conditions such as Crohn's disease [6], acute mesenteric ischemia [7], or gastrointestinal dysfunction [8]. Elevated d-lactate in non-intestinal related diseases have also been described, laying bare the specificity of this metabolite, like the detection of body fluids infections [9,10] or the prediction of 28-day mortality in critically ill septic shock patients [11]. These applications have recently been reviewed and the lack of knowledge of this metabolite compared to its levogyre counterpart l-lactate has been emphasized [1]. This insufficient material is partially due to the difficulty of performing a reliable d-lactate assay, especially in a high-volume rendering context on a 24-h basis. Indeed, most of the major clinical chemistry suppliers do not offer d-lactate assay on routine analysers. Until today, most available lactate-stamped assays have actually been l-Lactate assays, with either a spectrophotometric technique using l-lactate dehydrogenase or an electrochemical method using l-lactate oxidase. As a consequence, measuring d-lactate in biological fluids relies on slow rate semi-automated chemistry analysers using ELISA kits or, more expensively, on High-Performance Liquid Chromatography (HPLC) with Fluorescence Detection or Liquid Chromatography – Tandem Mass Spectrometry (LC-MS/MS) methods [[12], [13], [14]]. In addition, these two last methods both imply the acquisition of a chiral column, which is not frequently used otherwise. Here, we here propose an open channel analysis on Roche Cobas® 8000 c502 allowing a 24/7 rendering of plasma d-lactate. This method is a two steps end point enzymatic assay based on the reaction of d-lactate interconversion to pyruvate catalysed by D-LDH with a reduction of an NAD coenzyme to NADH. Detection is spectrophotometric at 340 and 700 nm along with the appearance of NADH (see technical sheet for details at www.biosentec.fr/docs/Tech/079-Kit-D-lactate-21031.pdf). BioSenTec (Portet-sur-Garonne, France) provided d-lactate enzymatic reagent kit and calibrator as well as d-lactate internal quality control (IQC) (https://www.biosentec.fr; product number: 079). Calibration is multipoint (0 µmol.L^−1^ and 1390 µmol.L^−1^) and linear. Based on pathological values found in the literature, we performed a 1:5 dilution of the provided quality control ([d-lactate] = 2500 µmol.L^−1^) in the standard Roche 9% NaCl diluent (reference number 04489357) to prepare a medium (500 µmol.L^−1^) level of IQC. In addition, we prepared a supplemental IQC with two levels (a low at ∼180 µmol.L^−1^ and a high at 1200 µmol.L^−1^) from a ≥99% pure d-lactate powder provided by Sigma-Aldrich (Saint-Louis, Missouri, United-States, reference number 71716). In-board reagents and internal quality controls were found to be stable at least 15 days between +2 and + 8 °C (cut-off: <10% bias from initial value).

To validate the method, we first determined the limit of detection (LoD) with a blank test. LoD was calculated as recommended by the French accreditation committee (COFRAC, [15]) as 3 times the standard deviation of measurement on water. Thus, LoD was 0.02 µmol.L^−1^. We then determined the limit of quantification (LoQ) using Horwitz curve [16] and a range of plasma spiked with different d-lactate concentrations. LoQ was calculated to 40 µmol.L^−1^, as relative standard deviation (RSD) was <10% at this concentration. Within-run imprecision, defined by coefficient of variation (CV%), for low, medium and high levels of internal quality control was 1.98%, 1.24% and 0.87% respectively (n = 30) and between-run imprecision for the medium level was 2.21% (n = 8 on 11 weeks period). Robustness was evaluated using the ratio of between-run and within-run imprecision CVs as described by the French accreditation committee (COFRAC, [15]). The ratio is 1.63, which is satisfying (acceptable: <2). Upper limit of linearity was determined using increasingly spiked plasma. The method used was still linear at the highest concentration tested: [d-lactate] = 15 mmol L^−1^. Regarding interferences, we assessed analytical impact of hemolysis (H), lipemia (L), and bilirubin (I). For these three interfering substances, H, I and L indexes were measured by the Roche HIL test (reference number 07470045). Acceptance criteria was a ±10% maximal bias from the initial value. Hemolysis impact was evaluated by adding increasing concentrations of hemolysate obtained by osmotic choc of distilled water on erythrocytes to a d-lactate spiked plasma. Hemolysis did not interfere under a free plasmatic hemoglobin concentration of 300 mg dL^−1^. Lipemia was evaluated by adding increasing concentrations of 20% Intralipid®. Briefly, in our laboratory lipemia is scaled from “limpid” to “lactescent +++”, with the following L index ranges: limpid: L = 0 to 17, opalescent +: L > 17), opalescent ++: L > 31), opalescent +++: L > 51), lactescent +: L > 110), lactescent ++: L > 131 and lactescent +++: L > 350. Lipemia interfered above a lipemic index of 46, corresponding to an opalescent ++ sample. Bilirubin interference was evaluated by adding increasing bilirubin solution (Sigma-Aldrich, reference number 14370) to a d-lactate spiked plasma. Icterus was significant above a direct bilirubin concentration of 103 µmol.L^−1^. It is worth noting that interference bias level was linearly correlated with hemolysis level, but not with lipemia nor bilirubin. Interference of spectrophotometric d-lactate assays with high l-Lactate Dehydrogenase concentrations have been described, as it disrupts the NAD^+^/NADH balance [17,18]. Thus, we explored this interference as follows: We made pools of plasma leftovers from patients with increasing levels of L-LDH (60, 250, 500, 750, 1000, 1500 and 3000 IU.L^−1^). Each pool was split in two and spiked with the above mentioned d-lactate powder to reach 100 and 200 µmol.L^−1^ of d-lactate. Interference did not exceed 10% of d-lactate increase until a L-LDH level of 1000 IU.L^−1^, which is consistent with the literature. To be noted that to avoid such interference, it is possible to deproteinize samples with precipitation or ultrafiltration [14,19]. Nevertheless, it implies additional preanalytical steps which can be challenging in routine practice. Regarding the stability in plasma, plasma pools collected on lithium heparin tube (Beckton Dickinson Microtainer^MD^) with d-lactate levels from 100 to 500 µmol.L^−1^ were stored at 4 °C for 72h and d-lactate levels remained stable (<10% difference from the h0 determination). Finally, we tested D-LDH stereospecificity by measuring d-lactate in a l-lactate spiked NaCl solution. l-lactate did not interfere (cut-off: <10% of bias from initial value) with the D-LDH assay at the maximal concentration we tested: [l-lactate] = 15 mmol L^−1^. The data validating this method are summarized in Table 1.

**Table 1:** Validation of an open-canal automatized d-lactate method.

Criteria	Quality indicator	Result
*Measurement range*		
Limit of Detection (LoD)	Blank test	0.02 µmol.L^−1^
Limit of Quantification (LoQ)	<10% relative standard deviation (RSD)	40 µmol.L^−1^
Superior limit of linearity	Need for dilution at high-concentration	>15 mmol L^−1^
*Analytical performances*		
Within-run imprecision	Coefficient of Variation (CV%)	IQC_low_: 1.98%
		IQC_medium_: 1.24%
		IQC_high_: 0.87%
Between-run imprecision		IQC_medium_: 2.21%
Robustness	CV ratio	1.63
*Interferences*		
Hemolysis	±10% maximal deviation	300 mg dL^−1^
Icterus		103 µmol.L^−1^
Lipemia		L index of 47 (highly opalescent)
l-Lactate Dehydrogenase		1000 IU.L^−1^
Storage time		72 hours in plasma at 4 °C

A wide range of clinical applications of d-lactate measurements has been established in the literature. This metabolite could represent an interesting biomarker due to its great specificity of bacterial anaerobic glycolysis. Beyond the scope of underlying gastrointestinal diseases, it may find a place as a first line biomarker in bacteraemia or severe infections. Indeed, a plasmatic or body fluid d-lactate concentration available within an hour from admission could be a useful tool upstream of microbiological analyses (*e.g*. blood culture, gram staining result …). However, d-lactate is not yet a routine assay proposed by major suppliers. Commercials kits are available but rely on semi-automated methods such as multi-well plate immunoassay with a plate reading step. Thus, in order to propose a d-lactate assessment in a 24/7 basis, each laboratory must develop an open channel analysis on their chemistry analyzer. Here we propose a short turnaround time, cost-effective automated method for plasma d-lactate assessment on Roche's chemistry analysing system, which is widely spread. With this method, we hope to promote d-lactate utilization in clinical practice as a specific bacterial metabolism biomarker, even a low levels. We are confident that developing highly specific bacterial metabolite assay can bring new insights to the field of clinical chemistry and microbiology.

## Declaration of competing interest

All authors of PLM-D-22-00056_R1, Adrien Turban, Sophie Gaubert, David Luque-Paz, Céline René, Nicolas Collet, Maxime Pawlowski, Claude Bendavid and Charles R. Lefèvre, declare that they have no competing interest for this manuscript.

## Data Availability

Data will be made available on request.

## References

[1] Levitt M.D., Levitt D.G. (2020). Quantitative evaluation of D-lactate pathophysiology: new insights into the mechanisms involved and the many areas in need of further investigation. Clin. Exp. Gastroenterol..

[2] Mishra D., Banerjee D. (2019). Lactate dehydrogenases as metabolic links between tumor and stroma in the tumor microenvironment. Cancers.

[3] Monroe G.R., van Eerde A.M., Tessadori F., Duran K.J., Savelberg S.M.C., van Alfen J.C., Terhal P.A., van der Crabben S.N., Lichtenbelt K.D., Fuchs S.A., Gerrits J., van Roosmalen M.J., van Gassen K.L., van Aalderen M., Koot B.G., Oostendorp M., Duran M., Visser G., de Koning T.J., Calì F., Bosco P., Geleijns K., de Sain-van der Velden M.G.M., Knoers N.V., Bakkers J., Verhoeven-Duif N.M., van Haaften G., Jans J.J. (2019). Identification of human D lactate dehydrogenase deficiency. Nat. Commun..

[4] de Bari L., Atlante A., Armeni T., Kalapos M.P. (2019). Synthesis and metabolism of methylglyoxal, S-D-lactoylglutathione and D-lactate in cancer and Alzheimer's disease. Exploring the crossroad of eternal youth and premature aging. Ageing Res. Rev..

[5] Bianchetti D.G.A.M., Amelio G.S., Lava S.A.G., Bianchetti M.G., Simonetti G.D., Agostoni C., Fossali E.F., Milani G.P. (2018). D-lactic acidosis in humans: systematic literature review. Pediatr. Nephrol..

[6] Cai J., Chen H., Weng M., Jiang S., Gao J. (2019). Diagnostic and clinical significance of serum levels of D-lactate and diamine oxidase in patients with Crohn's disease. Gastroenterol. Res. Pract..

[7] Memet O., Zhang L., Shen J. (2019). Serological biomarkers for acute mesenteric ischemia. Ann. Transl. Med..

[8] Teng J., Xiang L., Long H., Gao C., Lei L., Zhang Y. (2021). The serum citrulline and D-lactate are associated with gastrointestinal dysfunction and failure in critically ill patients. Int. J. Gen. Med..

[9] Marcos M.A., Vila J., Gratacos J., Brancos M.A., Jimenez de Anta M.T. (1991). Determination of D-lactate concentration for rapid diagnosis of bacterial infections of body fluids. Eur. J. Clin. Microbiol. Infect. Dis..

[10] Gratacós J., Vila J., Moyá F., Marcos M.A., Collado A., Sanmartí R., Brancós M.A., Jimenez de Anta M.T., Muñoz-Gómez J. (1995). D-lactic acid in synovial fluid. A rapid diagnostic test for bacterial synovitis. J. Rheumatol..

[11] Sapin V., Nicolet L., Aublet-Cuvelier B., Sangline F., Roszyk L., Dastugue B., Gazuy N., Deteix P., Souweine B. (2006). Rapid decrease in plasma D-lactate as an early potential predictor of diminished 28-day mortality in critically ill septic shock patients. Clin. Chem. Lab. Med..

[12] Cevasco G., Piątek A.M., Scapolla C., Thea S. (2011). A simple, sensitive and efficient assay for the determination of d- and l-lactic acid enantiomers in human plasma by high-performance liquid chromatography. J. Chromatogr., A.

[13] Hasegawa H., Fukushima T., Lee J.-A., Tsukamoto K., Moriya K., Ono Y., Imai K. (2003). Determination of serum d-lactic and l-lactic acids in normal subjects and diabetic patients by column-switching HPLC with pre-column fluorescence derivatization. Anal. Bioanal. Chem..

[14] Larsen T. (2017). Fluorometric determination of d-lactate in biological fluids. Anal. Biochem..

[15] SH-GTA-04pdf https://tools.cofrac.fr/documentation/SH-GTA-04.

[16] Horwitz W. (1982). Evaluation of analytical methods used for regulation of foods and drugs. Anal. Chem..

[17] Martí R., Varela E., Segura R.M., Alegre J., Suriñach J.M., Pascual C. (1997). Determination of d-lactate by enzymatic methods in biological fluids: study of interferences. Clin. Chem..

[18] Herrera D.J., Morris K., Johnston C., Griffiths P. (2008). Automated assay for plasma D-lactate by enzymatic spectrophotometric analysis with sample blank correction. Ann. Clin. Biochem. Int. J. Lab. Med..

[19] Rasmussen R.W., Straarup D., Thorlacius-Ussing O., Handberg A., Christensen P.A. (2021). Fully automatic d -lactate assay using a modified commercially available method. Scand. J. Clin. Lab. Invest..

